# Clinical and Histologic Outcomes of Biologically Oriented Alveolar Ridge Preservation: A Prospective Observational Study

**DOI:** 10.1111/cid.70048

**Published:** 2025-05-02

**Authors:** Antonio Rapani, Leonardo Tonegato, Paolo Savadori, Rebecca Martini, Riccardo Pasquali, Matteo Zotti, Vanessa Nicolin, Federico Berton, Claudio Stacchi

**Affiliations:** ^1^ Department of Medical, Surgical and Health Sciences University of Trieste Trieste Italy; ^2^ Medical and Dental Department of Morphological Sciences Related to Transplant, Oncology and Regenerative Medicine University of Modena and Reggio Emilia Modena Italy

**Keywords:** alveolar ridge preservation, bone substitutes, post‐extractive ridge modifications

## Abstract

**Introduction:**

This study aimed to evaluate the efficacy of Biologically oriented Alveolar Ridge Preservation (BARP) in minimizing post‐extraction ridge modifications compared with unassisted socket healing.

**Methods:**

A prospective controlled observational study was conducted involving 30 patients requiring single‐rooted upper premolar extractions. Patients were divided into two groups: the test (15 patients), which underwent a ridge preservation procedure combining absorbable collagen sponge in the middle and apical third of the socket and collagenated xenogeneic bone substitute in the most coronal part (BARP), and the control (15 patients), which healed spontaneously. Soft tissue contour changes after 6 months were analyzed using digital impressions. Moreover, histomorphometric analysis of the regenerated tissue was performed in the test group.

**Results:**

BARP significantly reduced post‐extractive mucosal ridge modifications compared to the control group. Mean vertical shrinkage at the mid‐buccal part of the edentulous site was 1.61 ± 0.61 mm (BARP) vs. 2.51 ± 0.64 mm (control; *t*‐test, df = 28, *p* < 0.001), and mean horizontal reduction was 3.37 ± 0.63 mm (BARP) vs. 4.34 ± 0.48 mm (control; *t*‐test, df = 28, *p* < 0.001). Histomorphometric analysis of the regenerated tissue showed 39.7% ± 9.1% newly formed bone with minimal residual graft material (5.0% ± 5.4%).

**Conclusion:**

BARP technique effectively minimizes post‐extractive soft tissue contour modifications and supports natural bone regeneration, resulting in adequate bone dimensions for implant rehabilitation.

## 1 Introduction

Dental implant therapy is today a common and reliable procedure for replacing missing teeth [1, 2, 3]. However, inadequate residual bone crest width and height may often pose challenges, requiring additional bone regeneration procedures before or at the time of implant placement [4, 5, 6]. These measures are clinically challenging, time‐consuming, costly, and are associated with a significant short‐ and long‐term risk of complications [7, 8]. To prevent these issues, limiting post‐extractive resorption of the alveolar crest is a crucial point.

After tooth extraction, the walls of the socket undergo significant three‐dimensional resorption, leading to changes in the shape and contour of the hard and soft tissues of the alveolar ridge [9, 10, 11, 12, 13, 14, 15]. The outcomes of this physiological remodeling differ from one site to another and from person to person, depending on various systemic and local factors, such as the thickness of the facial alveolar bone wall and other features of the local or site‐specific phenotype [10, 16, 17].

Numerous techniques have been proposed in the literature to counteract these volumetric changes, commonly referred to as alveolar ridge preservation (ARP). ARP refers to procedures aimed at maintaining ridge volume or minimizing its reduction within the bone envelope present at the time of tooth extraction while promoting bone growth within the socket [18, 19]. ARP techniques typically involve placing a bone graft material into the socket, often combined with a barrier membrane or a soft tissue graft. ARP techniques have been demonstrated to be more effective than unassisted socket healing in maintaining alveolar ridge dimensions, but they showed limited efficacy on promoting new bone formation [20]. A recent network meta‐analysis found that most grafting materials, including commonly used xenografts and allografts, did not improve new bone formation compared to natural healing of the post‐extractive socket [21]. Bone grafts are intended to counteract volumetric alveolar ridge reduction and act as osteoconductive scaffolds, supporting early healing and promoting bone growth. Ideally, these grafts should be gradually replaced by new bone: however, slow‐resorbing grafts may delay bone regeneration and remodeling by leaving residual particles that can cause foreign body reactions and occupy space needed for new bone [22, 23, 24]. Conversely, fast‐resorbing grafts might promote new bone formation but often have poor mechanical properties and low volumetric stability [25]. Moreover, the use of a grafting material in ARP procedures can prevent up to 30% of volume reduction in the coronal third but has little to no effect on the middle and apical thirds of the socket [26].

In an attempt to overcome these limitations, a novel ARP technique called Biologically oriented Alveolar Ridge Preservation (BARP) has recently been proposed in the literature [27, 28]. This procedure involves the use of absorbable collagen sponges in combination with a xenogeneic bone substitute. Collagen sponges are used to fill the middle and apical portions of the socket, while the bone substitute is used to graft the most coronal 4–5 mm of the socket, avoiding overfilling. A final layer of collagen sponge is then placed on top and secured with a cross‐stitch suture to prevent graft dispersion and favor secondary intention wound closure [27].

The BARP technique aims to achieve the following objectives: (i) to promote natural bone formation in the middle and apical parts of the socket by avoiding interference from bone substitutes; (ii) only the coronal portion of the socket is grafted to counteract bone remodeling effectively, since this area is the most susceptible to bone resorption; (iii) to stabilize the clot and graft at the socket entrance, improving the healing process.

The aim of this prospective controlled observational study was to compare the three‐dimensional changes of the soft tissue contour 6 months after tooth extraction, using the BARP technique, with those observed in unassisted healing. Additionally, an evaluation of the histological and histomorphometric results of the sites treated with the BARP technique after 6 months of healing was conducted.

The null hypothesis of the study is that there is no difference in the mucosal ridge dimensions 6 months after tooth extraction, when unassisted healing is compared with BARP.

## 2 Materials and Methods

### 2.1 Study Protocol

The present prospective controlled observational study was reported in accordance with the Strengthening the Reporting of Observational Studies in Epidemiology (STROBE) guidelines. All procedures adhered strictly to the Declaration of Helsinki for research involving human subjects.

### 2.2 Selection Criteria

Patients requiring extraction of a single‐rooted upper premolar, with adjacent teeth present, followed by dental or implant‐supported prosthetic rehabilitation, were eligible to participate in this study.

Patients were consecutively enrolled, provided they met the following inclusion criteria:

- Age > 18 years;
- no contraindications for oral surgery and implant therapy [29];
- sufficient bone height at the extraction site for the placement of a standard implant (≥ 8.5 mm);
- healthy oral mucosa with at least 3 mm width of keratinized tissue;
- socket Type I Subclass B (extraction socket with intact buccal bone and buccal bone thickness < 1.0 mm) [30];
- written informed consent given.

Patients were excluded from the study in the presence of any of the following exclusion criteria:

- Uncontrolled diabetes (HBA1c > 7.5%);
- uncontrolled or untreated periodontal disease;
- allergy to equine collagen;
- pregnant or breastfeeding women;
- tooth extraction requiring mucoperiosteal flap elevation and/or ostectomy;
- absent or damaged buccal bone wall after tooth extraction;
- buccal bone wall thickness after tooth extraction ≥ 1 mm;
- presence of large periapical lesions (> 3 mm diameter);
- patient unable to fully comply with the study protocol.

### 2.3 Group Allocation

Treatment was determined based on the planned prosthetic treatment of the edentulous site. If the neighboring teeth required prosthetic rehabilitation, a fixed dental bridge was planned, and the site was allocated to the spontaneous healing group (control). Conversely, if the adjacent teeth were intact, a single implant was planned, and the site was allocated to the BARP group (test).

### 2.4 Clinical Procedures

Patients recruited for this study underwent a comprehensive clinical examination. This included assessing periodontal conditions using probing and periapical radiographs and evaluating occlusal relationships. One week before surgery, all patients received professional deplaquing and were instructed to use a 0.2% chlorhexidine digluconate mouthwash twice daily until the day of surgery.

**FIGURE 1:**
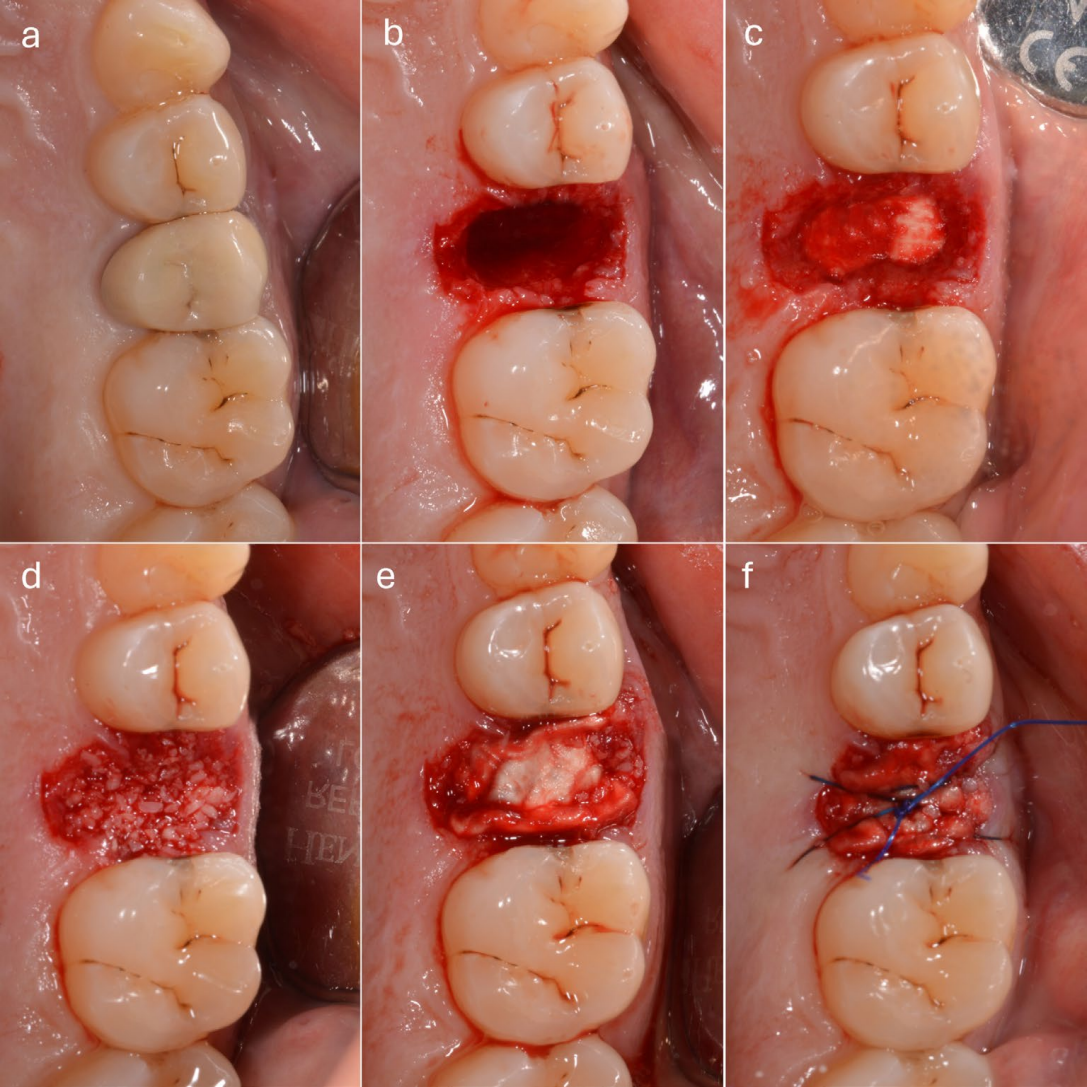
BARP clinical steps: (a) Pre‐operative situation with an endodontically hopeless second premolar; (b) flapless minimally traumatic tooth extraction was performed; (c) after socket debridement, a deep collagen layer was positioned; (d) xenogeneic bone substitute fills the most coronal 4–5 mm of the socket; (e) superficial collagen layer covering the xenograft; (f) collagen is stabilized with a cross‐stitch suture.

On the day of extraction (T0), a digital impression of the upper dental arch was taken with an intra‐oral scanner (iTero Element 5D, Align Technology, Tempe, AZ, USA). Then, after performing local anesthesia (2% mepivacaine HCl with 1:100 000 epinephrine), a flapless tooth extraction was performed, using levers only in the mesial and distal directions and forceps. After confirming the integrity of all socket walls using a periodontal probe, the gingival tissue was gently displaced by approximately 1 mm to allow direct contact between a bone caliper and the underlying buccal bone, enabling accurate assessment of its thickness (< 1 mm) without the need for flap elevation. BARP was carried out in the test group. After gently removing eventual intra‐alveolar granulation tissue, the post‐extraction socket was filled with an absorbable equine collagen sponge (Condress, Smith & Nephew, London, UK) up to 4–5 mm from the most coronal margin, providing support for the xenogeneic porcine cortico‐cancellous collagenated bone substitute in granules (Gen‐Os Osteobiol, Tecnoss, Giaveno, Italy), which was used to fill the socket completely, while taking care to avoid overfilling. An additional layer of absorbable collagen was applied over the bone substitute and stabilized with a cross‐stitch suture using non‐absorbable synthetic monofilament. No attempts were made to reach a primary intention closure (Figure 1).

In the control group, the extraction was performed using the same methods, but the socket was left to heal spontaneously.

Paracetamol (1000 mg tablets) was prescribed as needed.

Sutures were removed after 1 week, and the site was evaluated for healing and any complications. Patients underwent further evaluations at 2 weeks, 1 month, and 3 months after surgery to monitor soft tissue healing.

**FIGURE 2:**
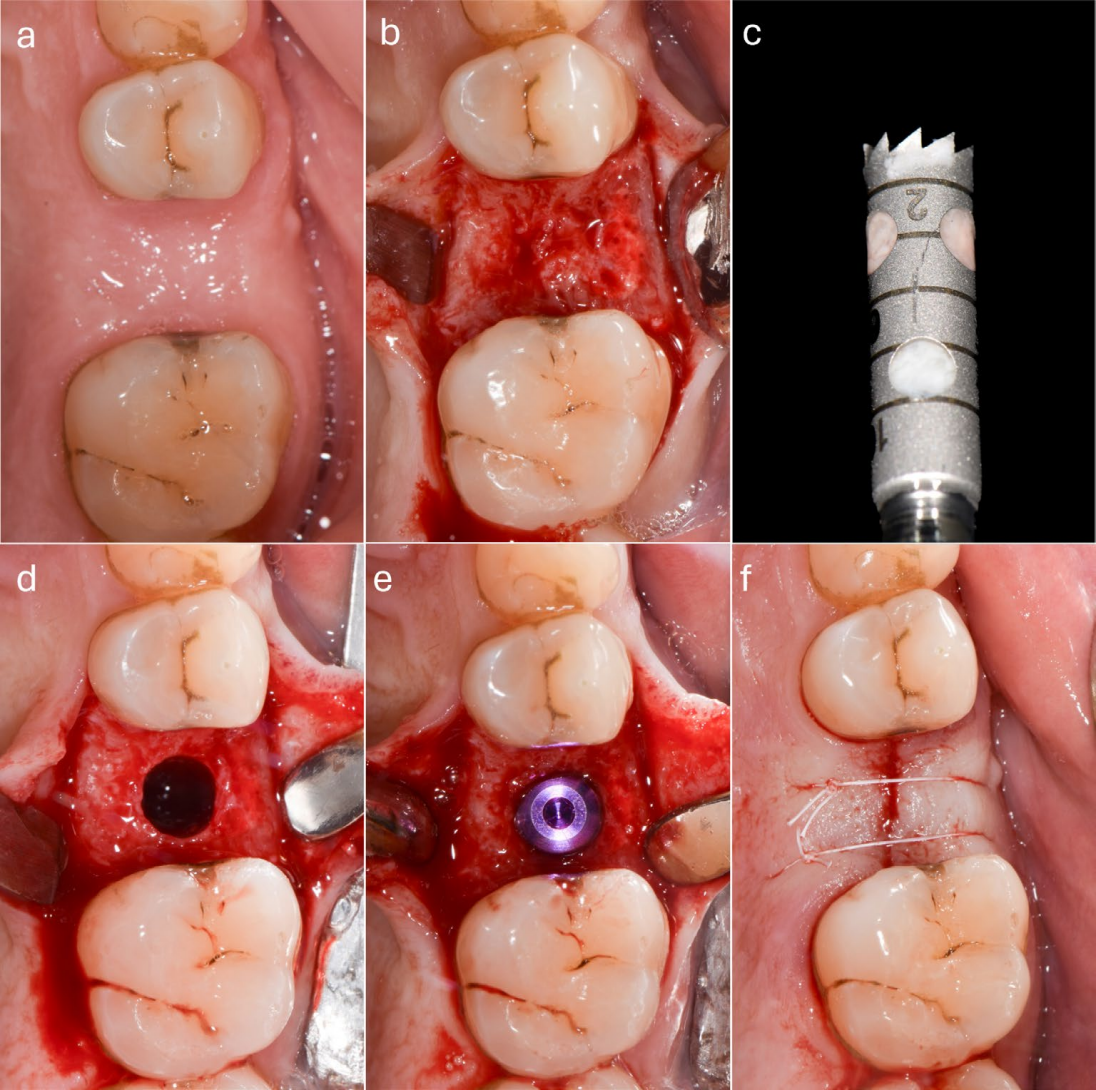
Surgical re‐entry 6 months after BARP: (a) Clinical aspect of the healed ridge; (b) clinical aspect of the healed bone crest; (c) bone biopsy taken at the planned implant site; (d) implant site preparation is finalized with specific drills; (e) implant seated in the final position; (f) flap closure for implant submerged healing.

At 6 months (T1), a digital impression of the upper dental arch was taken in both groups, and test group patients underwent implant placement. During implant site preparation, a bone biopsy was taken at the BARP site using a trephine drill with an inner diameter of 3 mm (2982.Y0.30, DenTag, Maniago, Italy) under copious irrigation (Figure 2).

### 2.5 Data Collection

Medical records were collected by an independent assessor (R.M.) and recorded in a specific case report form.

The following patient‐level information was gathered:

- age;
- gender;
- systemic diseases;
- medications;
- smoking habits;
- history of periodontal disease.

The following site‐specific data were collected:

- vertical dimensional changes of the soft tissue contour;
- horizontal dimensional changes of the soft tissue contour;
- newly formed bone (%NFB) at T1;
- newly formed non‐mineralized tissue (%NFNMT) at T1;
- residual grafting material (%RG) at T1.

## 3 Measurements of Soft Tissue Contour Variations

**FIGURE 3:**
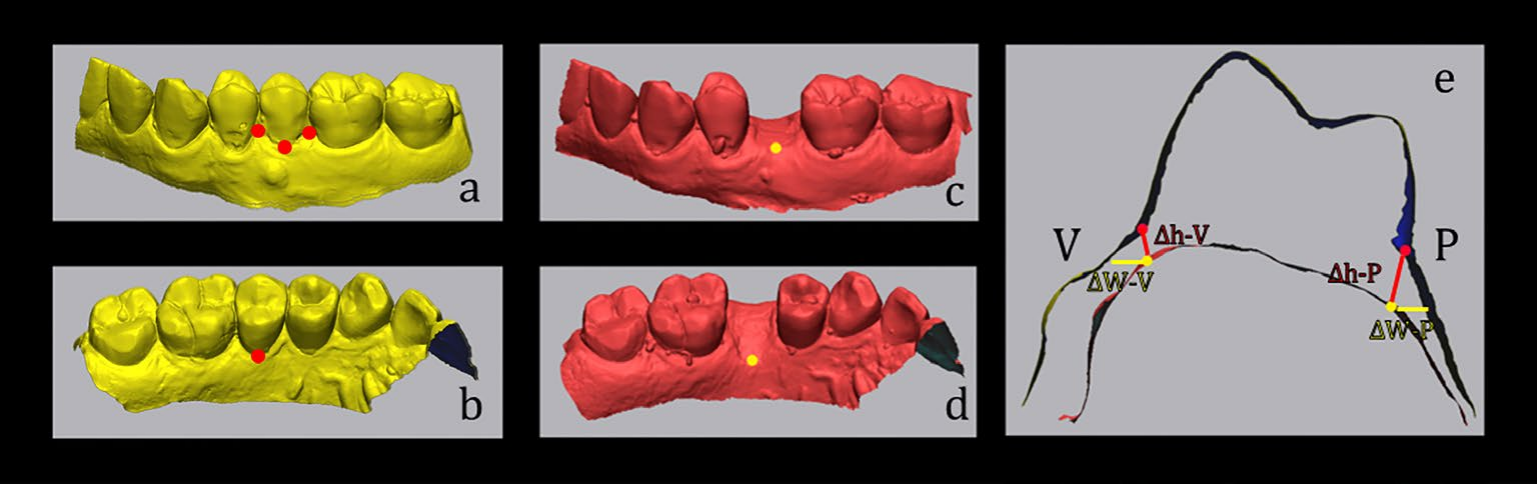
Reference points used to measure soft tissue contour modifications from T0 to T1: (a) Vertical resorption at mid‐buccal (ΔhV), interproximal mesial, and distal (ΔhM; ΔhD); (b) vertical resorption at mid‐palatal (ΔhP); (c) horizontal resorption at the buccal side (ΔWV); (d) horizontal resorption at the palatal side (ΔWP); (e) summary diagram.

Standard Tessellation Language (STL) files exported from digital impressions at T0 (before extraction) and T1 (after 6 months of healing) were superimposed using a metrology software (Geomagic Control X, Oqton, San Francisco, CA, USA) and linear dimensional changes were measured with a mean error of ±0.15 mm between STL files in adjacent untreated areas [31]. Vertical dimensional changes, both buccal and palatal, were assessed at the center of the mesio‐distal axis of the post‐extraction site, using the gingival zenith at T0 as a reference point (ΔhV; ΔhP). Vertical dimensional changes were also measured mesially and distally, with the gingival papilla at T0 as a reference point (ΔhM; ΔhD). Horizontal dimensional changes (on the buccal side, palatal side, and total) were evaluated at the center of the mesio‐distal axis of the post‐extraction site, using the most coronal point of the mucosal ridge at T1 as a reference point (ΔWV; ΔWP; ΔWtot) (Figure 3).

Measurements of soft tissue contour variations were performed by a calibrated trained operator (M.Z.). All measurements were repeated after 2 weeks following the same protocol, and the average of the two sessions was used in the final analysis. Intra‐rater reliability across the two measurement sessions was assessed using the intra‐class correlation coefficient (ICC) [32].

### 3.1 Histological and Histomorphometric Analyses

Histological and histomorphometric analyses were conducted by two of the authors (P.S. and R.P., respectively), remaining blinded to the study design and the source of the biopsies.

After harvesting, bone biopsies were kept inside the trephine drill to preserve the orientation of the bone core. The specimens were rinsed with a cold 5% glucose solution to remove blood residues while maintaining osmolarity. They were fixed in 10% buffered formalin (pH 7.2) for 3 days and then decalcified in 14% EDTA at pH 9 [33]. Subsequently, the samples were dehydrated in increasing concentrations of isopropyl alcohol, starting from 70% up to 100%, then cleared in xylene, and embedded in paraffin. Successive 4‐μm‐thick slices were cut from the resulting blocks and stained with hematoxylin and eosin trichrome stain [34].

Histomorphometric analysis was performed with a transmitted brightfield light microscope (Biostar B3, Exacta Labcenter, San Prospero, Italy) connected to a high‐resolution digital camera (Moticam 5.0, Motic, Xiamen, China). Image analysis was carried out using the image processing software Image‐Pro Plus 6.0 (Media Cybernetics, Rockville, MD, USA). Each biopsy was represented by three histological sections, and three rectangular microscopic fields of 3 × 4 mm were randomly selected from coronal, middle, and apical areas of each section. Tissue types NFB, NFNMT, and RG were quantified through a semi‐automatic segmentation process, with manual corrections made as needed under visual inspection. The data were expressed as the percentage of the area occupied by each selected tissue type relative to the total test area. The final result for each biopsy was the average of the three histological slices. The following histomorphometric parameters were measured or calculated: %NFB (NFB area per total area; NFB/TA), %NFNMT (NFNMT area per total area; NFNMT/TA), %RG (RG area per total area; RG/TA), and %TMT (total mineralized tissue: %NFB + %RG).

### 3.2 Outcome Variables

*Primary outcome measures*:

- vertical dimensional changes of the soft tissue contour from T0 to T1;
- horizontal dimensional changes of the soft tissue contour from T0 to T1.

*Secondary outcome measures*:

- %NFB after 6 months of healing in test sites;
- %NFNMT after 6 months of healing in test sites;
- %RG after 6 months of healing in test sites;
- total mineralized tissue (%TMT) after 6 months of healing in test sites;
- any complications or adverse events.

### 3.3 Sample Size Calculation and Statistical Power

The sample size of the present study was calculated using a statistical software (Primer of Biostatistics, Version 6.0, Mc Graw‐Hill, New York, NY, USA), based on data reported in a previous research on reduction in interproximal soft tissue height after maxillary single tooth extraction comparing ARP with unassisted healing (−1.0 ± 0.5 and −2.0 ± 0.9 mm, respectively) [35]. A sample of 11 patients for each group was required to detect significant differences between the two groups (5% confidence level with statistical power of 90%).

### 3.4 Statistical Analysis

All data were analyzed by an independent assessor (A.R.) using a statistical software (IBM SPSS Statistics for Windows, Version 26.0, IBM, Armonk, NY, USA). The patient was considered as the statistical unit (one extraction per patient). Descriptive statistics were expressed as mean with standard deviations for parametric data and as median (IQR) for non‐parametric data. The Shapiro–Wilk test was used to assess dataset distribution of the investigated outcomes at different time points. Differences in continuous variables between the two groups (test and control) were analyzed using an independent sample *t*‐test for parametric data and a two‐sample Wilcoxon rank‐sum test for non‐parametric data. A *p*‐value < 0.05 was considered statistically significant.

## 4 Results

### 4.1 Study Population

Thirty consecutive patients, selected from 52 initially screened at the Maxillofacial and Stomatology Clinic of Trieste University Hospital, were included in the present study. Fifteen patients (10 female, 5 male; mean age 55.4 ± 10.8 years, range 39–77) were assigned to the test group, while the remaining 15 patients (5 female, 10 male; mean age 62.1 ± 10.4 years, range 46–77) were assigned to the control group. Detailed demographic information is provided in Table 1. All surgeries were performed by the same operator (L.T.) between March 2024 and May 2024. No post‐operative complications occurred, and no drop‐outs were recorded during the entire study period.

**TABLE 1:** Demographic characteristics of the study sample.

	Control		Test		*p*
Gender	5 F (33.3%)	10 M (66.7%)	10 F (66.7%)	5 M (33.3%)	Chi‐square test with Yates correction: 0.144
Age	62.1 ± 10.4 years—range 46–77		55.4 ± 10.8 years—range 39–77		*t*‐test: 0.138
	Yes	No	Yes	No	
Smoking habits	7 (46.7%)	8 (53.3%)	2 (13.3%)	13 (86.7%)	Fisher's exact test: 0.109
History of periodontal disease	7 (46.7%)	8 (53.3%)	1 (6.7%)	14 (93.3%)	Fisher's exact test: 0.035^a^

Abbreviations: Control: unassisted socket healing; F: female; M: male; Test: biologically oriented alveolar ridge preservation (BARP).

^a^ Statistically significant.

### 4.2 Soft Tissue Contour Changes

#### 4.2.1 Vertical Variations

Mean vertical resorption from T0 to T1 at the mid‐buccal aspect of the mucosal ridge (ΔhW) was 1.61 ± 0.61 mm in the test group and 2.51 ± 0.64 mm in the control group (*t*‐test, df = 28, *p* < 0.001). Similarly, the vertical contraction of interproximal soft tissue was significantly lower when using BARP (ΔhM 0.92 [0.39] mm; ΔhD 0.72 [0.50] mm) than with unassisted healing (ΔhM 1.42 [1.19] mm; ΔhD 1.44 [0.71] mm) (Wilcoxon rank‐sum test; *p* < 0.01). In contrast, the mid‐palatal vertical dimensional change (ΔhP) did not show statistically significant differences between the two groups (test 1.45 ± 0.66 mm; control 1.84 ± 0.63 mm; *t*‐test, df = 28, *p* = 0.11).

#### 4.2.2 Horizontal Variations

Significant differences were observed between the two groups in horizontal shrinkage from T0 to T1 on both the buccal and palatal sides, as well as in the overall mean horizontal mucosal ridge reduction (test 3.37 ± 0.63 mm; control 4.34 ± 0.48 mm; *t*‐test, df = 28, *p* < 0.001). Complete results for vertical and horizontal mucosal ridge shrinkage are listed in Table 2.

**TABLE 2:** Comparison of ridge shrinkage from T0 to T1 between test and control groups (expressed in mm).

	Shapiro–Wilk test (*p*)	Control, mean ± SD/median (IQR)	Test, mean ± SD/median (IQR)	*p*	df
Δh‐V	0.575	2.51 ± 0.64	1.61 ± 0.61	< 0.001^a^	28
Δh‐P	0.678	1.84 ± 0.63	1.45 ± 0.66	0.11	28
Δh‐M	0.000	1.42 (1.19)	0.92 (0.39)	< 0.01^a^	n/a
Δh‐D	0.001	1.44 (0.71)	0.72 (0.50)	< 0.001^a^	n/a
ΔW‐V	0.037	2.24 (0.19)	1.94 (0.42)	< 0.001^a^	n/a
ΔW‐P	0.028	2.13 (0.31)	1.74 (0.57)	< 0.01^a^	n/a
ΔW‐Tot	0.542	4.34 ± 0.48	3.37 ± 0.63	< 0.001^a^	28

Abbreviations: Δh‐D: interproximal distal vertical ridge shrinkage; Δh‐M: interproximal mesial vertical ridge shrinkage; Δh‐P: mid‐palatal vertical ridge shrinkage; Δh‐V: mid‐buccal vertical ridge shrinkage; ΔW‐P: palatal horizontal ridge shrinkage; ΔW‐Tot: total horizontal ridge shrinkage; ΔW‐V: buccal horizontal ridge shrinkage; df: degrees of freedom; IQR: interquartile range; n/a: not available; SD: standard deviation.

^a^ Statistically significant.

The ICC scores indicated excellent intra‐examiner repeatability for soft tissue contour assessments (0.91; CI [0.87–0.95]).

### 4.3 Histological and Histomorphometric Evaluation

**FIGURE 4:**
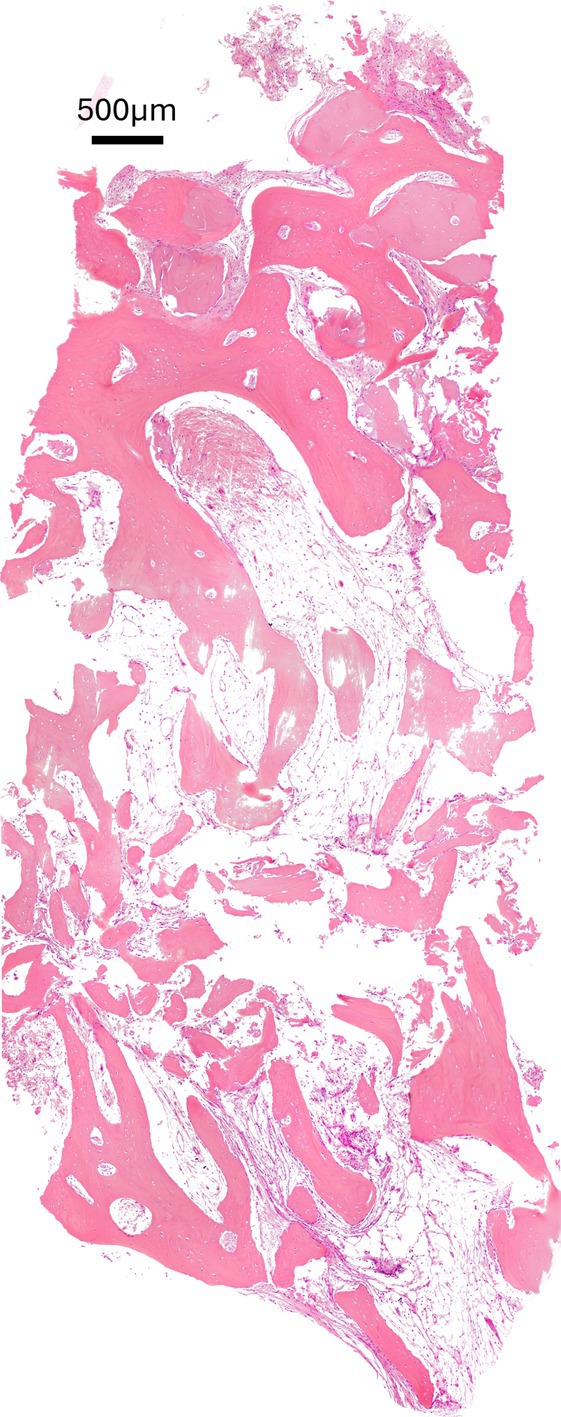
A representative biopsy of the newly‐formed tissue at T1 in BARP group shows that residual graft granules are present only in the most coronal portion of the sample, while in the middle and apical thirds, only newly‐formed bone tissue and marrow spaces are observed. Hematoxylin and eosin trichrome stain.

The morphological analysis of biopsies harvested from the test sites at T1 reveals the presence of NFB, connective tissue, and RG. Due to the specific characteristics of the BARP technique, the lower two‐thirds of the sample exhibit a complete absence of residual bone substitute (Figure 4).

**FIGURE 5:**
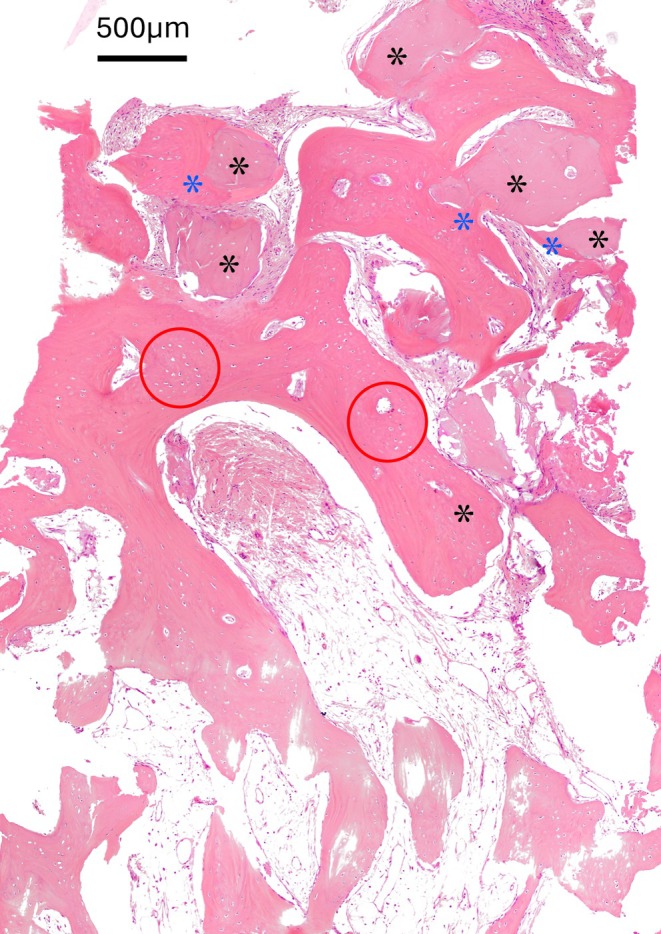
Residual graft granules (black asterisks) are in direct contact with the newly formed bone (blue asterisks), leading to the formation of bone bridges between particles and often resulting in their complete encapsulation. The presence of clustered osteocytes (circles) suggests that the newly formed bone is still undergoing reorganization and has not yet completed its remodeling and maturation processes. Hematoxylin and eosin trichrome stain.

RG granules appear in direct contact with the NFB, which creates bone bridges between the graft particles and often completely surrounds them. Multiple regions of maturing bone are evident, characterized by the presence of osteoid tissue and a slightly irregular Haversian system. These features suggest the development of secondary osteons resulting from bone regeneration and remodeling processes [36], indicating the early formation of a lamellar bone structure. The presence of clustered osteocytes suggests that the tissue is still undergoing reorganization and has not yet completed its remodeling and maturation processes (Figure 5).

**FIGURE 6:**
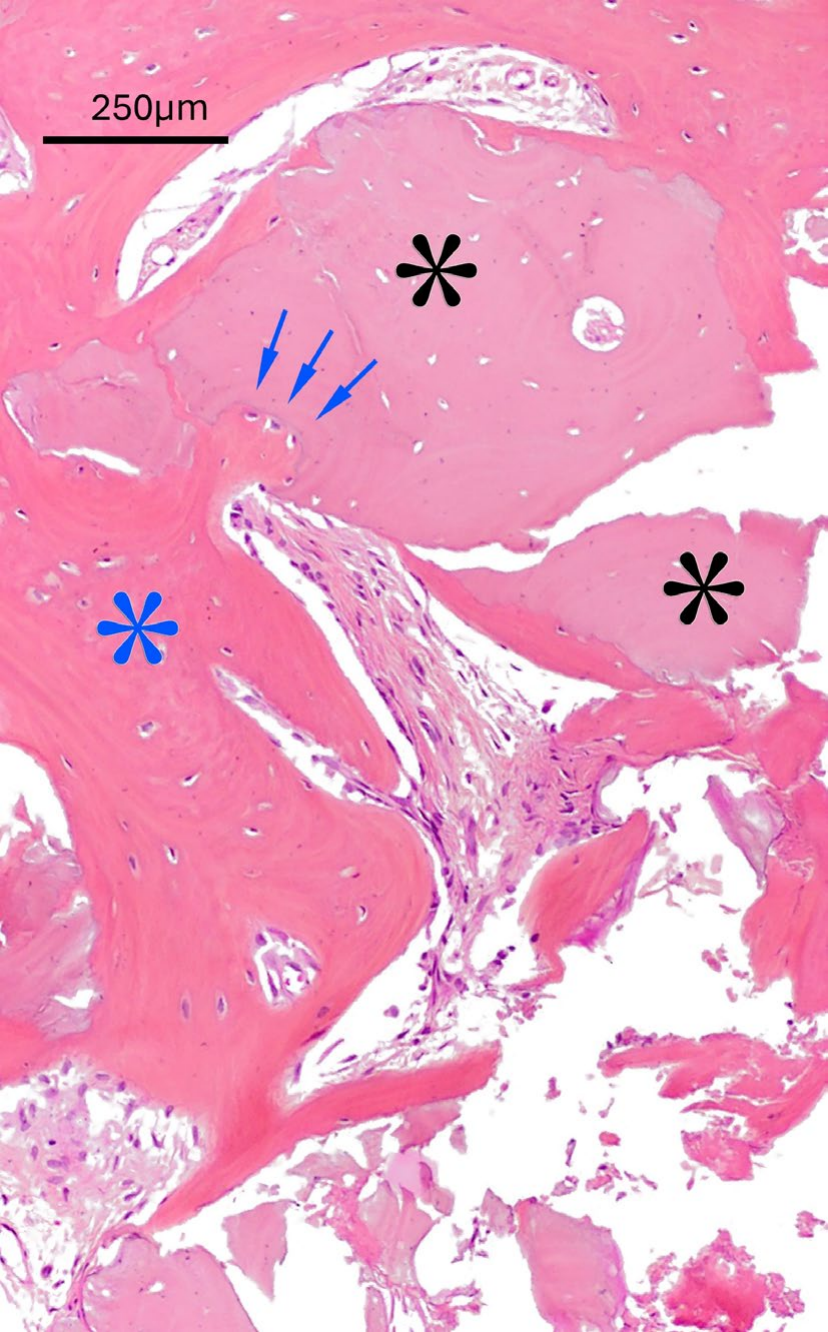
At higher magnification, a direct interface between the advancing bone formation front (blue asterisks) and the peripheral region of the residual graft (black asterisks) becomes evident, confirming excellent tissue integration of the bone substitute. In these areas, osteocytes are arranged in single‐cell cords forming a palisade‐like structure (arrows), encased by the portion of the bone trabecula currently undergoing growth. Hematoxylin and eosin trichrome stain.

At higher magnification, the direct relationship between the new bone formation front and the marginal portion of the residual bone substitute is also observed, demonstrating optimal tissue integration of the graft. Specifically, osteocytes are arranged in single‐cell cords forming a palisade, surrounded by the portion of bone trabecula undergoing growth (Figure 6).

The analyzed sections of the harvested samples exhibited a mean surface area of 11.44 ± 2.1 mm^2^. Histomorphometric analysis revealed a mean %NFB of 39.7 ± 9.1, %RG of 5.0 ± 5.4, and %NFNMT of 55.2 ± 10.4, with a cumulative mean %TMT of 44.7 ± 10.4.

## 5 Discussion

This prospective controlled observational study aimed to evaluate the efficacy of BARP compared to unassisted socket healing in preventing contraction of the mucosal ridge after 6 months of healing. Our results indicate that the BARP technique did not entirely prevent post‐extraction resorption, and a certain amount of bone loss can be expected during the healing process. Similar findings have been documented in previous systematic reviews on this topic, which demonstrate that no ARP technique or grafting material can completely eliminate post‐extractive modifications of the alveolar crest [19, 37].

However, the present findings lead to the rejection of the null hypothesis of the study, demonstrating a significant effect of the BARP technique in minimizing vertical and horizontal soft tissue contour modifications from T0 to T1 compared to unassisted socket healing. Mean vertical shrinkage of the mucosal ridge contour at the mid‐buccal, interproximal mesial, and distal aspects was significantly lower in the test group (1.61 ± 0.61; 0.92 [0.39]; 0.72 [0.50] mm, respectively) than in the control group (2.51 ± 0.64; 1.42 [1.19]; and 1.44 [0.71] mm, respectively). Similarly, the BARP group exhibited a mean horizontal shrinkage of the mucosal ridge of 3.37 ± 0.63 mm, with the control group showing a significantly greater mean ridge horizontal contraction (4.34 ± 0.48 mm; *p* < 0.001). In our opinion, even a dimensional reduction of approximately 1 mm may become clinically relevant, particularly in the esthetic zone or in sites with limited bone volume, where implant positioning and soft tissue outcomes are highly sensitive to underlying bone architecture. Such reduction might influence prosthetic planning or necessitate additional bone augmentation procedures. Overall, these findings are consistent with the results of quantitative analyses reported in previous systematic reviews evaluating the clinical outcomes of ARP performed using various approaches, including xenografts with socket seal, allografts with socket seal, or alloplastic materials with or without socket seal [19, 38, 39, 40, 41, 42, 43, 44]. However, even more noteworthy is the substantial alignment of the present results with the limited number of ARP studies that specifically analyzed cases with an intact buccal wall measuring less than 1 mm thickness at the time of extraction, as was done in this investigation [35, 45, 46, 47, 48]. In fact, the significant anatomical variability of extraction sockets can impact the regenerative potential of various proposed techniques to differing extents. Avila‐Ortiz et al. [44] highlighted that sites with a buccal bone thicker than 1.0 mm show significantly more favorable ridge preservation outcomes compared to those with a thinner buccal wall. Similarly, a recent decision tree proposed by Steigmann et al. [30] suggests that a thick, intact buccal bone (≥ 1 mm) enhances the socket's natural regenerative potential, allowing for optimal healing without the need for any supportive intervention.

Conversely, it is important to highlight that, in the present study, the mid‐palatal vertical dimensional change (ΔhP) from T0 to T1 did not differ significantly between the two groups (test: 1.45 ± 0.66 mm; control: 1.84 ± 0.63 mm; *p* = 0.11). The lack of significant differences in the palatal region may be attributed to its anatomical characteristics, including a more consistent blood supply, thicker fibromucosa, and denser cortical bone, which make this area less prone to resorption compared to the buccal side [11, 49].

A potential limitation of the present study is that evaluating soft tissue contour changes could underestimate the true protective effect of ARP on the bone crest. Although soft tissues often appear to reflect the shape of the underlying bone, this assumption is not fully supported by existing literature. Avila‐Ortiz et al. [48] noted that in many cases, particularly in spontaneous socket healing, soft tissue contour masked the actual degree of bone resorption. These findings are consistent with those of Chappuis et al. [50], who found no reliable correlation between hard and soft tissue changes and observed a compensatory soft tissue expansion into the bone compartment during unassisted socket healing.

Histomorphometric analysis of BARP sites at T1 showed a mean %NFB of 39.7 ± 9.1. This amount of NFB is notably greater compared to the data reported in a meta‐analysis examining histological and histomorphometric outcomes following ARP procedures performed using xenografts (23.6 [95% CI = 2.13–66.71]) [51]. This difference can be attributed to the selective grafting of the coronal portion of the socket, approximately 4–5 mm. This targeted approach in BARP preserves the physiological processes of natural bone deposition in the middle and apical regions, thereby facilitating optimal wound healing and tissue maturation [27, 28]. While a similar selective grafting approach has been described in the so‐called “Yin‐Yang” technique [52, 53], that method involves grafting approximately half of the socket and employs a collagen membrane for coverage. In contrast, the BARP protocol focuses solely on the coronal 4–5 mm of the socket, which corresponds to the most resorption‐prone region. This area is particularly susceptible due to its limited vascular supply, direct exposure to the oral environment, and its anatomical configuration as the thinnest portion of the alveolar crest—especially on the buccal aspect. Notably, this critical region does not necessarily coincide with the apical half of the socket, supporting the rationale for a more anatomically targeted grafting strategy. Additionally, BARP uses a collagen sponge rather than a membrane; while both materials support clot stability and soft tissue integration, the sponge represents a more cost‐effective and clinically efficient solution. For these reasons, the mean %RG measured in BARP sites at T1 (5.0 ± 5.4) is considerably lower compared to the %RG reported in the aforementioned meta‐analysis (37.14 [95% CI = 21.27–54.59]) for ARP procedures performed with xenografts.

Some other limitations should be considered when interpreting these findings. First, the study's sample size was relatively small, which may limit the statistical power to detect subtle differences in some outcomes. While the sample size was adequate for primary outcomes, a larger cohort would provide a more robust validation of the present results. Second, the study was conducted in a controlled clinical setting with a specific patient population. The exclusion criteria, such as uncontrolled systemic diseases and intact buccal bone wall, may restrict the generalizability of the findings to other populations, particularly those with more complex medical or anatomical conditions. Third, the study sample showed no significant differences between the test and control groups in terms of age, gender, and smoking habits, ensuring a balanced comparison. However, the significant difference between groups in the history of periodontitis may have impacted healing outcomes, serving as a potential confounding factor that should be accounted for in the interpretation of results. Patients with a history of periodontitis often present with an altered local environment, including chronic inflammation, reduced bone quality, and compromised regenerative capacity, which may negatively affect both soft and hard tissue healing after tooth extraction. This is supported by previous evidence suggesting that periodontally compromised sites show less favorable outcomes following ARP procedures compared to periodontally healthy sites [38, 44]. Fourth, the 6‐month follow‐up period, though standard for evaluating ARP outcomes, may not fully capture long‐term bone remodeling or the possibility for further graft resorption over time.

Finally, as mentioned before, measurements were based on soft tissue contours, which may not always accurately reflect underlying bone changes. Future studies including CBCT also for the control group, which were not possible in the present study for ethical reasons, may provide a more detailed assessment of post‐extractive bone remodeling.

## 6 Conclusion

The BARP technique effectively minimizes post‐extraction soft tissue contour modifications compared to unassisted socket healing at maxillary sockets with intact, thin buccal plate. Histomorphometric evaluation of BARP sites revealed a high percentage of NFB with minimal RG material confined to the coronal third of the socket. In the remaining regions, natural bone formation was actively promoted. The clinical findings indicate that the BARP technique is a reliable and efficient method for preserving alveolar ridge dimensions and ensuring sufficient bone for subsequent implant placement, reducing the need for additional regenerative procedures.

## Author Contributions

**Antonio Rapani:** conceptualization (equal), formal analysis (lead), methodology (equal), software (lead), validation (lead), writing – review and editing (equal). **Leonardo Tonegato:** data curation (equal), investigation (lead), writing – review and editing (equal). **Paolo Savadori:** data curation (lead), investigation (equal); writing – review and editing (equal). **Rebecca Martini:** data curation (equal), visualization (lead), writing – review and editing (equal). **Riccardo Pasquali and Matteo Zotti:** data curation (equal), investigation (equal), writing – review and editing (equal). **Vanessa Nicolin:** project administration (lead), resources (equal), writing – review and editing (equal). **Federico Berton:** conceptualization (equal), methodology (equal), software (equal), validation (equal), writing – review and editing (equal). **Claudio Stacchi:** conceptualization (lead), investigation (equal), methodology (lead), supervision (lead), writing – original draft (lead), writing – review and editing (equal).

## Ethics Statement

The study protocol had been approved by the relevant ethical committee (Comitato Etico Unico Regionale Friuli Venezia Giulia, no. 2024‐OS‐47) and was registered in a public clinical trials database (www.clinicaltrials.gov‐NCT06546826).

## Consent

Written informed consent was obtained from each participant before enrollment in the study. Patients were assured that their data would be used anonymously for research purposes only.

## Conflicts of Interest

The authors declare no conflicts of interest.

## Data Availability

The data supporting the findings of the present study are available from the corresponding author upon reasonable request.
